# The Mysterious Letter

**Published:** 1870-04

**Authors:** 


### HALL'S

### JOURNAL OF HEALTH.

Vol. XVII. APRIL, 1870.

### THE MYSTERIOUS LETTER.

The following letter was found to-day on our office table. The name and home of the writer are alike unknown to us.

"Did you ever hear of any lady who did not like compliments? Now please excuse me for claiming *all those* in your letter, though *not* intended for me. I claim all the honor of having originated and carried into effect our 'Dr. Hall Reading Club.' And the enthusiasm with which I attempted and carried it out, was my reward; my husband knew nothing of it till I had finished my work, and paid the bill myself, — not in opposition, however, to the opinion of my husband, for he, as well as myself, are delighted readers of all the works that we have seen of Dr. H. Let me add here, too, that I met with a very hearty response to my proposition for a Club, and one object in fixing the sum at 17 cents, was to let a larger number see the *Journal*, and with the hope that many of them will feel that they must have an entire copy for themselves. It is my purpose to introduce this plan into our neighboring towns, and I sincerely wish that you may be obliged to issue many thousand copies.

" Pray do not set me down as an egotist, because of this letter. My reason is this. It is the case with *all* clergymen of limited means, to procure *all* their *reading* matter for their *own* use, and we poor ministers' wives and daughters *starve* for the want of literature not *wholly* clerical. Science and art, and even the standard poets, are denied us. This may influence you in the choice of the 'other reading matter,' which you so kindly promised to send, and which may interest our ladies at our 'Club meetings.' I invited them to our house last week, with a view to organizing the Club, and we had a very crowded room, and the reading was spicy. Now, dear sir, if you will pardon *this* trespass upon your time, any business letters hereafter shall be more brief."

The Editor purposes to write a book, entitled, MOTHERHOOD, OR PARENTAL IMPRESS ON THE MENTAL, MORAL, AND PHYSICAL CONSTITUTION OF THE CHILD. The Preface will read, perhaps, as follows: —

The great end of human life is to secure an existence beyond the grave, high, holy, happy, and immortal.

The great wisdom of human life, is to do all that is possible to attain that existence, each for himself.

The great beneficence of human life, is to do all that can be done, consistently with other duties, to help one another to attain an object of such infinite importance, especially the children born to us.

How to perform the work, and when to begin it, will be the object of the book to point out, in the clearest, plainest way possible.

HELP ONE ANOTHER

is the spontaneous aspiration of every noble nature, of every generous bosom. The great ensample of all ages, in a work so literally god-like, is found in Him " who died that we might live," and they are best entitled to kinship with Him, who most closely follow His footsteps.

The aims and ambition of the world seem to be changing in these later days; aforetime, the money-hunter and the man-hunter seemed to loom up before the vision of the people of all ages, when there were only two heroes — of the battle field and the bank; a Napoleon who killed most men; a Rothschild who gathered most money, each working for his own individual self. Since Bonaparte's time, no one has aspired to be a world's conqueror. " Peace " is the motto of all nations; of all nations it is the prayer at this hour. Peace promotes science, art, mechanism, and the accumulation of large fortunes; while the increased thrift which long peace gives any community, affords the members of it more time for reflection, and for the contemplation of the highest truths which can engage the human mind. The promise now is, that henceforth there will be

TWO CONQUERING HEROES, the man who can get the most money, and do the most good with it, and the man who can do the most good by individual, personal effort; and we must say, this latter is the grandest example of a high humanity. Some give all their time, some give all their money to the one great end of

### HELPING THE HELPLESS TO HELP THEMSELVES.

In their different lines come Howard, Nott, Peter Cooper, Dorothea L. Dix, George Peabody, William A. Cornell, Daniel Drew, A. T. Stewart, and a multitude of less noted names, rated at the court of heaven not by the number of the dollars given, but by the purity of the motive and the heartiness of the deed.

It may be said with great truthfulness, that no city on the globe, large or small, gives near as much money or personal effort towards ameliorating the condition of man, as New York; there are men and women who give every year a large portion of their whole available time, and others who give one, two, ten, fifty thousand dollars a year, towards purposes of benevolence, only to be known when they are dead and gone. There are ladies and gentlemen known to us, who, when they are not often enough called on for contributions, make it a point to hunt out persons who know where help is needed, or can find out, and say to them, "Here is some money. I wish you would place it where it will help somebody." We personally know a gentleman who is the almoner of some of the rich, who go to him, hand him their money, take no receipt, ask for no vouchers, because they have the most implicit confidence that every cent will be conscientiously and judiciously appropriated. And the delightfulness of the thing is, that the distributor is never happier than when he is at such work. And it is no new thing with him; he has grown gray in his labor of love, and has grown young at it, too! At the age of threescore years and ten, his step is elastic, his eye bright, his form erect, and his whole countenance beams with pleasurableness. He makes no show or parade; if you follow him, you may find out what he does; as for himself he tells nobody; we have never known him to be sick an hour, not a minute; and day or night, summer or winter, rain or hail, sleet, storm, or snow, it's all the same; he is always ready to take his staff and go out on his mission of mercy and of love; and now, after seventy years of habit, he could not help doing good to somebody. A day's do-nothing would be the death of him. His end will be to die with his armor on, or maybe he will be "translated," as Enoch of the olden time.

Like him is our correspondent; and may be she, in time, will be equal to him,

A GOOD WORKER

in the great vineyard of humanity. This working for others, how it wakes up the pulses of the heart, how it imparts health to the body and happiness to the mind! A long lease of life will be given as the earthly reward of such industries.

THE HELPING HAND.

Only think of a Society of such a name; what a wide sweep of operations it would have; into its capacious folds could be taken members from all classes, climes, and creeds, constituting a universal

BROTHERHOOD OF GOOD DOING, with a motto of one sentence, a creed of one article, a confession of faith of two words, a banner of but one inscription, short, compact, comprehensive; which, wherever it met the eye, would make the whole heart tingle with delight; and which would draw from its deepest depths a joyous responsiveness which would happify the whole being. Reader! what say you to carrying this banner aloft, from this good hour, and resolve to continue it until you die? But would you like to know what the inscription is, before you make the promise? It is plain English: the veriest child would comprehend its fullest meaning, the instant the eye fell upon it; and is only this and no more:—

HELP ONE ANOTHER.

The very words themselves would have a humanizing influence, would tend to soften the whole heart of humanity, and inspire the noblest of all ambitions.

The best kind of "help," that which has no debasement in it, is to put it in a fellow-creature's power to help himself, and then forever after he will feel himself a man; such an one will always rise, and can never come to destitution, degradation, and crime. In another direction we can "help" others, that is to say, to be

RELIGIOUS, by setting them a good example, by talking with them, and giving them things to read which will draw their attention to the subject. Suppose you give the books which you have read, and will probably never read again, to poor boys, or women, or men who have not the means of purchase, and would greedily devour them.

NEWSPAPERS, periodicals, magazines, and pamphlets, — we read them over, are very much pleased with one or more articles, then carefully lay them away, with the full intention of looking at them again; but there are hundreds, and thousands, and unnumbered millions of pages of most excellent and instructive matter put away on shelves, in barns, cellars, garrets, in dwellings all over the land, which have never been disturbed since the day they were laid away, five, fifteen, fifty years ago, and never will be. What an immeasurable waste of power for good is here; one, two, ten, or ten thousand other heads might have been enlightened as much as ours was at the first perusal; as many hearts might have been as much delighted, as many pleasant pastimes might have been enjoyed, if these papers and magazines had been cast upon the waters and allowed to drift to some humble

COTTAGE BY THE SEA.

It does seem to be almost a sin to take up a religious newspaper of the day, devour it ourselves, and then lay it away, on the almost impossible contingency that we might want to read it again.

How carefully we put aside our magazines, and agricultural weeklies and monthlies, with their wealth of information and instruction, with the intention of having them bound one day. In what countless thousands of cases the day for binding never comes. And for the one case in a thousand in which they are bound, are they ever taken from the shelves again? Let the reader answer the question from his own personal experience. Let another course be pursued from this hour. Read your paper or periodical as carefully as you may; but as soon as the next one comes, give it to some neighbor, or friend, or laborer, or servant, or little boy, and just imagine his satisfaction when he takes it to his humble home, and spreads it out before the fire, or on the table; how he pores over it, and spells out the big words; and how the family begin to talk of what he reads; or again, what a pleasant remembrance it would be to mail it postpaid to some friend in the country, or far away, who would recognize the handwriting, and thus have the joy of reading it doubled. Who shall deny that if such a disposition were made of our newspapers, and magazines, and pamphlets, instead of stowing them away to be dusted, and smoked, and worm-eaten, multitudes would not be made happier and better, and ourselves none the losers and none the worse.

### DIFFUSING HEALTH.

Another way of doing good is to impart information to the ignorant as to the importance of taking care of the health, of avoiding disease and thus cutting off the cause of a large share of the sorrows and sufferings of our kind. Our lady correspondent has adopted a means of doing good, by the institution of a

### READING CLUB, which, if followed, — and it can be done, — in every town, and village, and neighborhood in our country, would have an effect for good absolutely incalculable. A club of ten, twenty, or more persons could meet once or twice or oftener in a week, for purposes of reading, conversation, and remark, which could be made a source of enjoyment, improvement, cultivation, and good feeling to every member. A religious newspaper could be read one night, the *Journal of Health* another, some monthly magazine a third, with intervals for remark, inquiry, suggestion, argument, interchange of views, and drawing out sentiments. This would be a means of promoting conversational power, of giving fluency of language, and of sharpening the wits for reply, less formal than in the debating society, and certainly much more efficient and instructive.

There are scores of thousands of persons in the United States who, if properly approached by a friend, would cheerfully subscribe for our *Journal*, and who, in a single year's reading, would gather ideas in reference to health and disease, which would be of life-long advantage; would thereby be saved from many a sick hour, and perhaps from premature death. Let us all then

HELP ONE ANOTHER

To get along in the world,
To live healthfully,
To get to heaven.

